# Pentobarbital Coma With Therapeutic Hypothermia for Treatment of Refractory Intracranial Hypertension in Traumatic Brain Injury Patients: A Single Institution Experience

**DOI:** 10.7759/cureus.10591

**Published:** 2020-09-22

**Authors:** Jacob E Bernstein, Hammad Ghanchi, Samir Kashyap, Stacey Podkovik, Dan E Miulli, Margaret Rose Wacker, Raed Sweiss

**Affiliations:** 1 Neurosurgery, Riverside University Health System Medical Center, Moreno Valley, USA; 2 Neurosurgery, Arrowhead Regional Medical Center, Colton, USA

**Keywords:** pentobarbital, traumatic brain injury, hypothermia

## Abstract

Introduction

Traumatic brain injury (TBI) results in primary and secondary brain injuries. Secondary brain injury can lead to cerebral edema resulting in increased intracranial pressure (ICP) secondary to the rigid encasement of the skull. Increased ICP leads to decreased cerebral perfusion pressure which leads to cerebral ischemia. Refractory intracranial hypertension (RICH) occurs when ICP remains elevated despite first-tier therapies such as head elevation, straightening of the neck, analgesia, sedation, paralytics, cerebrospinal fluid (CSF) drainage, mannitol and/or hypertonic saline administration. If unresponsive to these measures, second-tier therapies such as hypothermia, barbiturate infusion, and/or surgery are employed.

Methods

This was a retrospective review of patients admitted at Arrowhead Regional Medical Center from 2008 to 2019 for severe TBI who developed RICH requiring placement into a pentobarbital-induced coma with therapeutic hypothermia. Primary endpoints included mortality, good recovery which was designated at Glasgow outcome scale (GOS) of 4 or 5, and improvement in ICP (goal is <20 mmHg). Secondary endpoints included complications, length of intensive care unit (ICU) stay, length of hospital stay, length of pentobarbital coma, length of hypothermia, need for vasopressors, and decompressive surgery versus no decompressive surgery.

Results

Our study included 18 patients placed in pentobarbital coma with hypothermia for RICH. The overall mortality rate in our study was 50%; with 60% mortality in pentobarbital/hypothermia only group, and 46% mortality in surgery plus pentobarbital/hypothermia group. Maximum ICP prior to pentobarbital/hypothermia was significantly lower in patients who had a prior decompressive craniectomy than in patients who were placed into pentobarbital/hypothermia protocol first (28.3 vs 35.4, p<0.0238). ICP was significantly reduced at 4 hours, 8 hours, 12 hours, 24 hours, and 48 hours after pentobarbital and hypothermia treatment. Initial ICP and maximum ICP prior to pentobarbital/hypothermia was significantly correlated with mortality (p=0.022 and p=0.026). Patients with an ICP>25 mmHg prior to pentobarbital/hypothermia initiation had an increased risk of mortality (p=0.0455). There was no statistically significant difference in mean ICP after 24 hours after pentobarbital/hypothermia protocol in survivors vs non-survivors. Increased time to reach 33°C was associated with increased mortality (r=0.47, p=0.047); with a 10.5-fold increase in mortality for >7 hours (OR 10.5, p=0.039).

Conclusion

Prolonged cooling time >7 hours was associated with a 10.5-fold increase in mortality and ICP>25 mmHg prior to initiation of pentobarbital and hypothermia is suggestive of a poor response to treatment. We recommend patients with severe TBI who develop RICH should first undergo a 12 x 15 cm decompressive hemicraniectomy because they have better survival and are more likely to have ICP <25 mmHg as the highest elevation of ICP if the ICP were to become and stay elevated again. Pentobarbital and hypothermia should be initiated if the ICP becomes elevated and sustained above 20 mmHg with a prior decompressive hemicraniectomy and refractory to other medical therapies. However, our data suggests that patients are unlikely to survive if there ICP does not decrease to less than 15mmHg at 8 and 12 hours after pentobarbital/hypothermia and remain less than 20 mmHg within first 48 hours.

## Introduction

Traumatic brain injury (TBI) results in primary and secondary brain injury with primary brain injury occurring due to mechanical damage during the initial trauma and can result in various injuries such as skull fractures, subdural hematomas, epidural hematomas, cerebral contusions, and traumatic subarachnoid hemorrhage [1]. Secondary brain injury occurs after the initial injury and is due to oxidative stress, ischemic injury, inflammation, and other systemic or cerebral factors that can lead to further neurological injury [2]. Neurological critical care treatment strategies are directed towards preventing and treating secondary brain injury [1,2]. Secondary brain injury can cause cerebral edema resulting in increased intracranial pressure secondary to the rigid encasement of the skull. Increased intracranial pressure (ICP) leads to decreased cerebral perfusion pressure which leads to cerebral ischemia [2]. Intracranial pressure >20 mmHg has been found to be associated with poor neurologic outcomes with a historical range of elevated ICP of 15-25 mmHg [1,2]. Management of traumatic brain injury includes a combination of surgical and medical management directed at reducing intracranial pressure, improving cerebral oxygenation, and cerebral blood flow [1,2]. According to the 2016 Brain Trauma Foundation guidelines, ICP monitoring is recommended for the management of patients with severe TBI to reduce in-hospital and two-week post-injury mortality (Level II B recommendation) [3]. Prior recommendations from 2007 recommended ICP monitoring for patients with Glasgow coma scale (GCS) <8 with intracranial pathology [4]. Treatment of elevated ICP includes such therapies as head elevation, straightening the neck, analgesia, sedation, cerebrospinal fluid (CSF) drainage, mannitol and/or hypertonic saline administration [1-5]. Refractory intracranial hypertension (RICH) occurs when the above fail to control the elevated ICP (>20 mmHg) [1,4]. Next line medical therapy includes the initiation of a pentobarbital coma which acts to lower ICP, reduce cerebral blood flow, reduce cerebral oxygen consumption, reduce cerebral oxygen metabolism, and decrease neuro-excitotoxicity by reducing the release of glutamate and aspartate in brain tissue [1,6,7]. Pentobarbital administration has a level IIB recommendation from the traumatic brain injury guidelines for use in controlling elevated ICP refractory to maximum standard medical and surgical treatment [3]. Decompressive craniectomy has also been shown to decrease ICP in cases of TBI with RICH [8-10]. In animal models, therapeutic hypothermia has been shown to decrease secondary injury through reducing cerebral metabolic demands, inflammation, excitotoxicity, lipid peroxidation, and cell death [11]. It has been shown to decrease ICP and improve outcomes in adults in some studies [12,13]. However, three large multicenter randomized controlled studies have failed to show a benefit of hypothermia (33 °C) in TBI [14-16]. Therapeutic hypothermia has been shown to have worsening outcomes with an increase in mortality in children [11,17]. In adults, data are conflicting and currently the use of therapeutic hypothermia is not currently recommended for prophylactic or routine use [3,18]. A single-center randomized controlled trial with 82 total patients with severe TBI showed an improvement in functional outcomes in patients with initial GCS of 5-7 treated with 24 hours of hypothermia (33 °C) initiated 10 hours after injury [13]. However, following this was a large multi-center randomized controlled trial with a total of 392 patients with severe TBI treated with hypothermia (33 °C) for 48 hours within 6 hours from injury. This trial failed to show an improvement in patients treated with hypothermia compared to normothermia and was stopped early due to futility [15]. Patients in the hypothermia group also had more complications [15]. Therefore, hypothermia is not standard of care for patients with severe TBI and is still under investigation. At this time, no studies have looked at the use of pentobarbital and therapeutic hypothermia together for the management of RICH or in combination of decompressive craniectomy. The goal of this study is to further investigate the safety and effectiveness of concomitant use of pentobarbital coma and therapeutic hypothermia for the treatment of refractory intracranial hypertension.

## Materials and methods

Methods

This was a retrospective review of a prospectively collected database of patients admitted to the neurosurgery service at Arrowhead Regional Medical Center for traumatic brain injury who developed refractory intracranial hypertension requiring placement into a pentobarbital-induced coma with therapeutic hypothermia from 2008 to 2019. Patients were included in the study if they were at least 15 years of age, experienced a traumatic brain injury, and were placed in pentobarbital-induced coma with hypothermia for refractory intracranial hypertension. We defined refractory intracranial hypertension as intracranial pressure >20 mmHg for >30 minutes despite head elevation, straightening of the neck, analgesia, sedation, CSF drainage, mannitol and/or hypertonic saline administration. This is in contrast to the Eisenberg et al. study in 1988 that randomized patients into conventional medical therapy versus barbiturate therapy in patients without a craniectomy if there ICP was >25 mmHg for 30 min, >30 mmHg for 15 min, and >40mmHg for 1 min. If patients had a prior craniectomy they were included if ICP was >15 mmHg for 15 min, >20mmHg for 10 min, and >30 mmHg for 1 min [6]. Primary endpoints included mortality and good recovery which was designated at Glasgow outcome scale (GOS) of 4 or 5, and improvement in intracranial pressure. Secondary endpoints included any adverse events, length of ICU stay, length of hospital stay, length of pentobarbital coma, length of hypothermia, time to 33°C from initiation of pentobarbital coma, need for vasopressors, complications, and decompressive surgery versus no decompressive surgery needed.

Pentobarbital/hypothermia protocol

All patients in this protocol were on mechanical ventilation with a central venous access (for infusion of vasopressors if needed), arterial line (for hemodynamic monitoring), and external ventricular drain (for intracranial pressure monitoring and CSF drainage). All patients were placed on continuous electroencephalogram (EEG) for monitoring of burst suppression. All patients given a pentobarbital loading dose of 2.5 mg/kg intravenous (IV) every 15 minutes for four doses, then 5 mg/kg/hr IV drip for three hours, then 2 mg/kg/hr continuous IV drip. The pentobarbital drip was then titrated 1-3 mg/kg/hr to maintain burst suppression (one to two burst per page) on continuous EEG. The pentobarbital loading dose was often started after EEG ordered but prior to the EEG being connected in order to begin emergent treatment. Patients were placed in therapeutic hypothermia with the use of the Artic Sun cooling blanket (Bard Medical, Covington, GA). The patients core body temperature was lowered to and maintained at 33°C for a minimum of 24 hours but typically remained until ICP was consistently <20 mmHg [19]. Blood cultures were drawn every morning to monitor for occult infection given immunosuppression that can occur with pentobarbital use and a lack of febrile response with induced hypothermia. A complete metabolic panel was collected every 6 hours to monitor for any metabolic derangements that can occur due to pentobarbital and hypothermia use. Post pyloric feeding tubes were placed to reduce the incidence of ventilator-associated pneumonia as per brain trauma guidelines [3]. All patients were placed on an extensive bowel regimen consisting of docusate sodium, bisacodyl, and fleet enemas. When patients are coming out of the medically-induced coma, their core body temperature was increased by 0.1°C every hour to reduce the risk of rebound cerebral edema and intracranial hypertension [20]. Pentobarbital was incrementally weaned off once a patient’s temperature reached 37 °C.

Surgery

Surgery was performed on admission for evacuation of traumatic hematomas if patients met criteria based on the guidelines for surgical management of traumatic brain injury [21, 22, 23]. In these cases, the decision of performing a craniectomy versus craniotomy was based on presence of diffuse injury such as traumatic subarachnoid hemorrhage, cortical contusions, and amount of intraoperative cerebral edema observed. Furthermore, in patients with RICH, a decompressive craniectomy was performed for patients with new or worsening midline shift >5 mm and signs of herniation such as unilateral pupillary dilation. A large unilateral 12 x 15 cm frontotemporalparietal decompressive craniectomy was the preferred technique as recommended in the traumatic brain injury guidelines [3].

## Results

In our study, 18 patients with severe traumatic brain injury (GCS 3T-8T) were placed into a pentobarbital coma with hypothermia protocol as previously described. Thirteen patients underwent decompressive craniectomy. Of these, ten patients underwent surgery prior to placement in pentobarbital/hypothermia protocol and eight of these patients underwent a primary decompressive craniectomy on hospital day (HD) one for evacuation of hematomas with mass effect. Two patients had a secondary decompressive craniectomy on HD three for ICP>20 mmHg for 30 minutes despite first-tier medical management (Table 1). Three patients who’s ICP were initially controlled on pentobarbital and hypothermia required secondary decompressive craniectomies (on HD 6, HD 16, HD 18) due to uncontrolled ICP>20mmHg for >30 minutes. Five patients with diffuse brain injury and ICP>20 mmHg for >30 despite first-tier medical management, underwent pentobarbital coma and hypothermia protocol without undergoing surgery. Ten patients underwent a unilateral decompressive craniectomy (hemicraniectomy), one patient underwent a bilateral frontotemporalparietal craniectomies, and two patients underwent a bifrontal decompressive craniectomy and one of those patients pathology consisted of severe bifrontal contusions. The overall mortality rate in our study was 50%; with a 60% mortality rate in pentobarbital/hypothermia without surgery group and 46% mortality rate in surgery plus pentobarbital/hypothermia group. The maximum ICP prior to pentobarbital/hypothermia was significantly lower in patients who had a prior decompressive craniectomy than in patients who were placed into pentobartital/hypothermia protocol first (28.3 vs 35.4, p<0.0238). There was a lower mortality trend in patients who received a decompressive craniectomy than those that did not; however, this was not statistically significant.

**Table 1:** Patient demographics, mortality, surgery, and other data ICP, intracranial pressure; ICU, intensive care unit; GCS, Glasgow coma scale; GOS, Glasgow outcome score; T, intubated; HD, hospital day

Patient demographics, mortality, surgery, and other data	
Number of Patients	18
Age	27.5 (15-53)
Sex	14 Males: 4 Female
Mechanism	Motor vehicle accident-6 Motorcycle accident-2 Auto vs pedestrian-1 ATV(All-terrain vehicle) accident-1 Fall-5 Gunshot wound-2
Decompressive Craniectomy	13 Patients; 8 on HD 1 prior to Pentobarbital/Hypothermia, 2 on HD 3 prior to Pentobarbital/Hypothermia, 3 after placement on Pentobarbital/Hypothermia (HD 6, 16, 18)
Mean GCS on Admission	5.4 (3T-14)
Mean GCS prior to Pentobarbital coma/Hypothermia	5.3 (3T-8T)
Mean GCS on Discharge	4.8
Mortality	50%
Mortality without surgery	60%
Mortality with Surgery	46%
Mean Initial ICP (mmHg)	17.3
Mean ICP prior to Pentobarbital Coma/Hypothermia (mmHg)	31.4
Mean Maximum ICP 24 hours after Pentobarbital Coma/Hypothermia (mmHg)	22.8
Mean time to Start Pentobarbital Coma/Hypothermia (days)	2.72
Mean Time to reach 33 Celsius (hours)	8.7
Mean Length of Pentobarbital Coma (days)	11.4
Mean Length of Hypothermia (days)	9.5
Mean Length of ICU Stay (days)	38.6
Mean Length of Hospital Stay (days)	39.6
Mean Glasgow Outcome Score	2 (1-5)

At three-month follow-up, two patients had a good recovery (GOS 4 or 5), three patients with a severe disability (GOS 3), and four patients were in a vegetative state (Table 2). There were no significant difference in Glasgow outcomes scores at three months in patients that had surgery compared to those that did not have surgery.

**Table 2:** Glasgow outcomes scores at three months GOS, Glasgow outcome score

	Surgery (13)	No Surgery (5)
GOS 1 (Death)	6	3
GOS 2 (Vegetative State)	3	1
GOS 3 (Severe Disability)	3	0
GOS 4 (Moderate Disability)	0	0
GOS 5 (Good Recovery)	1	1

Placement into the pentobarbital/hypothermia protocol significantly reduced ICP at 4 hours, 8 hours, 12 hours, 24 hours, and 48 hours after treatment (Table 3). Initial ICP and maximum ICP prior to pentobarbital/hypothermia protocol were significantly lower in survivors versus non-survivors (Table 4). The initial ICP and maximum ICP prior to pentobarbital coma/hypothermia were shown to be significantly correlated with mortality (r=0.53, p=0.022 and r=0.53, p=0.026, respectively). Patients with an ICP>25 mmHg prior to pentobarbital/hypothermia initiation had an increased risk of mortality (Chi-square= 4, p =0.0455) (Table 5). There was no statistically significant difference in ICP 24 hours after pentobarbital/hypothermia protocol in survivors versus non-survivors (Table 4). ICP at 4, 8, 12, 24, and 48 hours after pentobarbital and hypothermia were weakly correlated with mortality, although non-significantly (r=0.16, 0.25,0.25, 0.39, and 0.40). There was a non-significant trend towards increased mortality in patients with an ICP >15mmHg at 8 (62%) and 12 (71%) hours after pentobarbital and hypothermia. A maximum ICP of >20 mmHg within 24 and 48 hours after treatment was also associated with an increase in mortality, although this was not statistically significant. There were medium negative correlations with initial ICP and GOS, maximum ICP prior to pentobarbital/hypothermia and GOS, and ICP prior to discharge and GOS (Pearson r=-0.45, -0.44, and -0.46); these were not statistically significant. There was no correlation with the length of hypothermia or pentobarbital and GOS. For the induction of hypothermia, the mean time to reach 33°C was 8.7 hours. Increased time to reach 33°C was associated with increased mortality (r=0.47, p=0.047); with a 10.5-fold increase in mortality for >7 hours (OR 10.5, p=0.039).

**Table 3:** ICP prior to pentobarbital/hypothermia and after treatment ICP, intracranial pressure

	ICP (mmHg)	P Value
Maximum ICP Prior to Pentobarbital/Hypothermia	31.4	None
Mean ICP @ 4 hours after Pentobarbital /Hypothermia	17.7	P=0.0001
Mean ICP @8 hours after Pentobarbital/Hypothermia	15.4	P<0.0001
Mean ICP @ 12 hours after Pentobarbital/Hypothermia	13.8	P<0.0001
Mean ICP @ 24 hours after Pentobarbital/Hypothermia	22.8	P=0.0055
Mean ICP @ 48 hours after Pentobarbital/Hypothermia	22.5	P=0.0033

**Table 4:** Intracranial pressure among survivors versus non-survivors ICP, intracranial pressure

Intracranial Pressure (mmHg)	Survivors	Non-Survivors	P value
Initial ICP (mean)	11.8	22.7	0.0198
Maximum ICP prior to Pentobarbital/Hypothermia (mean)	27.7	35.1	0.0167
Maximum ICP 24 hrs after Pentobarbital/Hypothermia (mean)	18.8	26.8	0.0889

**Table 5:** Mortality and ICP prior to pentobarbital/hypothermia ICP, intracranial pressure

ICP (mmHg)	Dead	Alive	Number of Patients	Mortality
ICP >25 mmHg prior to Pentobarbital/Hypothermia	8	4	12	66%
ICP 25 or less prior to Pentobarbital/Hypothermia	1	5	6	17%
Chi Square=4, p=0.0455	9	9	18	

Vasopressor support was required in 83% of patients while on the pentobarbital/hypothermia protocol in order to maintain a cerebral perfusion pressure goal of 60 mmHg-80 mmHg. Ventilator-associated pneumonia was the most common complication (55%) followed by ileus in 22% (Table 6).

**Table 6:** List of complications

Complications	Number of Complications
Ventilator/Hospital-Acquired Pneumonia	10
Ileus	4
Acute Respiratory Distress Syndrome	2
Renal Failure	2
Sepsis	2
Deep Vein Thrombosis	1
Ventriculitis	1

## Discussion

In severe TBI, refractory intracranial hypertension can occur despite the best medical and surgical management. In these cases, the mortality and morbidity are high. Patients in our study were placed into a pentobarbital-induced coma with hypothermia with or without decompressive surgery. The overall mortality rate in our study was 50%; only 11% (22% of survivors) of patients had a good recovery (GOS 4 or 5). Our mortality rate is similar to Marshall et al, who had a cohort of 55 patients with RICH who underwent pentobarbital coma with a mortality rate of 64% but with 70% of survivors having a good functional recovery (GOS 4 or 5) [1]. Hypothermia was not used in the Marshal *et al.* study. The use of hypothermia in TBI is controversial. Hypothermia has been shown to be beneficial in animal models and in a randomized controlled clinical trial (RCCT) in 1997 but has been shown to be harmful in children and ineffective in adults in three recent large multicenter randomized clinically controlled trials when compared to standard medical care [3,11,14-18]. However, these studies have not looked at the combined use of hypothermia with pentobarbital-induced coma as in our study. In our study, we found a significant correlation with increased time to reach 33°C; with a 10.5-fold increase in mortality for >7 hours. Thus, we recommend starting the hypothermia process as soon as possible while pentobarbital infusion is initiated with a goal of reaching 33°C in less than seven hours. 

Pentobarbital-induced coma has been shown to lower ICP, reduce cerebral blood flow, reduce cerebral oxygen consumption, reduces cerebral oxygen metabolism, and decrease neuroexcitotoxicity by reducing release of glutamate and aspartate in brain tissue [1,6,7]. Prophylactic pentobarbital use has not been found to be beneficial and its use in patients not requiring surgery has been shown to be harmful secondary to causing hemodynamic instability [24,25]. The use of pentobarbital does come with serious risks including severe hypotension, increased incidence of pneumonia and sepsis, and decreased gastrointestinal motility [1,24,25]. Treatment with hypothermia has also been shown to induce hypotension [26]. While hypotension can lead to cardiac complications, it also leads to decreased cerebral blood flow and cerebral perfusion pressure. In our study, 83% of patients required vasopressor support while on pentobarbital to maintain a cerebral perfusion pressure (CPP) of 60 mmHg to 80mmHg. On average our patients required vasopressors for eight days and averaged use of 2.5 vasopressor agents. No patients died as a result of hypotension leading to cardiac arrest. In our study protocol, we routinely obtain daily chest x-rays to monitor for infiltrates and place post-pyloric feeding tubes which have been shown to decrease the risk of ventilator-associated pneumonia [3]. Despite this effort, ventilator-associated pneumonia occurred in 55% of patients. Also, despite its risk of use, our study supports prior evidence that pentobarbital is effective in lowering intracranial pressure in patients with RICH after TBI [1,6,7]. Initial ICP and maximum ICP prior to pentobarbital/hypothermia was significantly correlated with mortality and lower in survivors than non-survivors as seen in Table 3, and were negatively correlated with GOS, although not statistically significant. This indicates that patients with lower initial ICP and lower ICP prior to pentobarbital coma with hypothermia have better outcomes. Patients with a maximum ICP >25 mmHg had a 66% mortality versus 17% mortality for patients with maximum ICP <25 mmHg prior to initiation into pentobarbital/hypothermia (Chi-square 4, p =0.0455). Patients with an ICP >30mmHg prior to pentobarbital and hypothermia had a 75% mortality and patients with ICP >30 mmHg had a 25% mortality, although this was not statistically significant. Based on our results an ICP of >25 mmHg is suggestive of a patient having a poor outcome and pentobarbital coma with hypothermia may not be effective in these patients.

Decompressive surgery has historically had better-reported survival in patients with RICH after TBI than medical care alone with reported mortality rates of <30% [1]. The Decompresive Craniectomy in Diffuse Traumatic Brain Injury (DECRA) trial compared bifrontotemporoparietal craniectomy versus standard medical care for treatment of RICH and found that bifrontotemporoparietal craniectomy significantly reduced mean intracranial pressure, duration of ventilator support, and length of ICU stay but with no difference in mortality while having a worse functional outcome at six months [10]. One hypothesis for the worse functional outcome is axonal stretching that could occur after decompression [10]. The medical care group included the use of hypothermia and barbiturates but did not specify treatment protocols or how many patients were treated with hypothermia and barbiturates [10]. The Trial of Decompressive Craniectomy For Traumatic Intracranial Hypertension (RESCUEicp) study compared decompressive craniectomy versus medical management for RICH in which 87% of the medical group received an unspecified barbiturate infusion with a median time of 53 hours [25]. Patients in both groups received therapeutic hypothermia (23.3% in surgical and 27% in medical) prior to randomization into surgery versus medical care groups [25]. The surgery group had a significantly lower mortality rate (26.9% vs 48.9%) but had higher rates of vegetative state, lower severe disability, and upper severe disability. The surgery group was shown to have higher rates of favorable outcomes at 12 months [25]. Our study was retrospective in nature with a review of patients with RICH who were placed into a pentobarbital coma with hypothermia and thirteen patients underwent decompressive craniectomy with eight patients developing RICH after having a decompressive craniectomy for the initial trauma and thus requiring placement in pentobarbital coma with hypothermia. In the eight patients who had surgery on admission, four survived. Two patients had a decompressive craniectomy on HD three and were later placed in pentobarbital/hypothermia; both survived. Three patients required a decompressive craniectomy after pentobarbital/hypothermia for uncontrolled ICP with one patient surviving. Patients who had surgery in addition to pentobarbital and hypothermia had a trend towards improved survivability although it was not statistically significant.

Treatment recommendations

For induction of hypothermia, we recommend starting the hypothermia process as soon as possible while pentobarbital infusion is initiated with a goal of reaching 33°C in less than seven hours.

**Figure 1:**
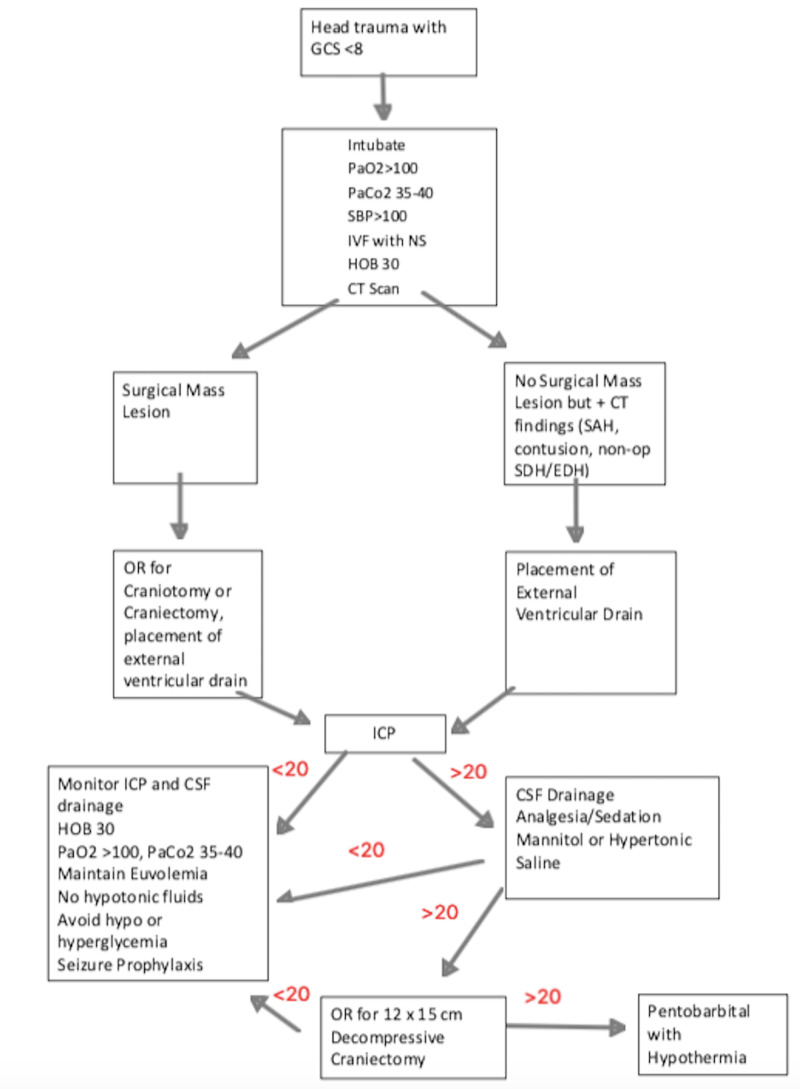
Treatment recommendations HOB, head of bed; ICP, intracranial pressure, ICP in units of mmHg; CT, computed tomography; SAH, subarachnoid hemorrhage; SDH, subdural hematoma; EDH, epidural hematoma; IVF, intravenous fluids; NS, normal saline; OR, operating room; CSF, cerebrospinal fluid; GCS, Glasgow coma scale

Of the 10 patients who underwent surgery prior to pentobarbital/hypothermia protocol, four patients died, and six patients had a maximum ICP of <25 with one death in the group. Our results indicate that the maximum ICP prior pentobarbital and hypothermia protocol is significantly correlated with mortality while ICP of 25mmHg prior to pentobarbital and hypothermia significantly associated with improved survivability (Table 4). The maximum ICP prior to pentobarbital/hypothermia was significantly lower in patients who had a prior decompressive craniectomy than in patients who were placed into pentobarbital/hypothermia protocol first (28.3 vs 35.4, p<0.0238). Thus, we recommend that for patients with RICH, they should first undergo a 12 x 15 cm decompressive craniectomy for ICPs >20 mmHg. If the ICP continues to be uncontrolled (>20mmHg) then they should be placed in a pentobarbital coma with hypothermia (Figure 1). However, our data suggest that if the ICP does not decrease to less than 15 mmHg at 8 and 12 hours after treatment and remain less than 20 mmHg within the first 48 hours, then they are unlikely to survive.

Limitations

One limitation of our study was that there was not a direct comparison of surgery versus pentobarbital with hypothermia for ICH. Furthermore, our study is limited by the small sample size, disproportionate number of male to female patients, lack of control group, and retrospective nature. Future studies involving a randomized controlled study comparing surgery versus pentobarbital-induced coma with concurrent hypothermia and comparing pentobarbital with hypothermia versus pentobarbital alone would be beneficial in elucidating effectiveness of the procedures. Another limitation is follow-up time; we were only able to account for a three-month follow-up period to ascertain GOS. It is possible that at six months or one-year post-injury that the patients could have continued to improve.

## Conclusions

Refractory intracranial hypertension can develop in patients with severe traumatic brain injury and is associated with high mortality and morbidity. Pentobarbital-induced coma with hypothermia significantly lowers intracranial pressure but may require vasopressor support for hemodynamic stability and maintenance of adequate cerebral perfusion pressures. Delays in initiating hypothermia induction should be avoided as increased time to reach 33°C is significantly correlated with mortality; with a 10.5-fold increase in mortality for >7 hours. The initial ICP and maximum ICP prior to pentobarbital/hypothermia initiation is correlated with increased mortality. Patients with initial ICP’s <25 mmHg and maximum ICP <25 mmHg prior to pentobarbital infusion have better outcomes. Thus, an ICP >25 mmHg is suggestive of poor response to pentobarbital coma with hypothermia. We recommend patients with severe traumatic brain injury who develop refractory intracranial hypertension should first undergo a 12 x 15 cm decompressive craniectomy because they have better survival and are more likely to have ICP <25 mmHg if the ICP were to become and stay elevated again. Pentobarbital and hypothermia should be initiated if the ICP becomes elevated and sustained above 20 mmHg with a prior craniectomy. However, our data suggest that patients are unlikely to survive if their ICP does not decrease to less than 15 mmHg at 8 and 12 hours after treatment and remain less than 20 mmHg within the first 48 hours.
